# Effectiveness and safety of electroacupuncture and its cotreatment with electronic moxibustion in the treatment of patients with moderate benign prostatic hyperplasia using alpha blocker: Study protocol for an assessor-blinded, randomized controlled clinical trial

**DOI:** 10.1097/MD.0000000000019678

**Published:** 2020-04-10

**Authors:** Hyo Bin Kim, Young Il Kim, Ju Hyun Jeon, Eunseok Kim, Jin Youp Kim, Ojin Kwon, Young Eun Choi, Changsop Yang, Chang-Hyun Han

**Affiliations:** a Department of Acupuncture and Moxibustion Medicine, College of Korean Medicine, Daejeon University, Daejeon; b Department of Clinical Korean Medicine, Graduate School, Kyung Hee University, Seoul; c Clinical Medicine Division, Korea Institute of Oriental Medicine; d Korean Convergence Medicine, University of Science and Technology (UST), Campus of Korea Institute of Oriental Medicine, Daejeon, Republic of Korea.

**Keywords:** benign prostatic hyperplasia, electroacupuncture, electronic moxibustion, randomized controlled trial, study protocol, traditional medicine

## Abstract

**Background:**

Benign prostatic hyperplasia (BPH) is a medical condition that affects the quality of life by causing lower urinary tract symptoms (LUTS) in 40% to 70% of men aged ≥60 years. Medication treatment is primarily recommended for patients with BPH if their symptom score based on the International Prostate Symptom score (IPSS) is above the moderate level. However, electroacupuncture (EA) and electronic moxibustion (EM), one of the most recent complementary and alternative treatments, are suggested as adjuvant treatments in the improvement of LUTS caused by BPH with respect to the limitations of medication treatments, such as side effects or no improvement in LUTS despite treatment. Therefore, this study aimed to evaluate the effectiveness and safety of EA and its cotreatment with EM for the improvement of LUTS in patients diagnosed with BPH using an alpha blocker but with moderate symptoms on the basis of IPSS.

**Methods/design:**

This protocol is a 2-arm parallel-design, randomized, controlled assessor-blinded clinical trial. Seventy-eight patients diagnosed with BPH are randomized to one of the following groups: [EA and its cotreatment with EM + alpha blocker group] and [alpha blocker group]. [EA and its cotreatment with EM + alpha blocker group] continues to use the previously prescribed alpha blocker and visits the study institution 3 times a week for 6 weeks to receive the cotreatment of EA and EM. [Alpha blocker group] continues to use the previously prescribed alpha blocker for 6 weeks. To evaluate the effectiveness of the EA and its cotreatment with EM, the followings are measured: total score of the IPSS, IPSS quality of life assessment, EuroQol-Five dimension, maximum and average urinary flow rate (Qmax and Qave), and prostate size at the baseline, 3rd, 6th, and 12th weeks. The primary effectiveness endpoint measures the average change in the total score of the IPSS at the 6th week. Side effects are recorded at each visit.

**Discussion:**

The results of this study are expected to provide useful information on the effectiveness and safety of the EA and its cotreatment with EM for patients with BPH with regard to the improvements in LUTS.

**Trial registration:**

Clinical Research Information Service of Republic of Korea (CRIS-KCT0004411), October 31, 2019.

## Abstract 2

Supplemental Digital Content is available in the text

## 1 Introduction

Benign prostatic hyperplasia (BPH) is a condition that is closely related to aging and male hormones and affects the quality of life by causing lower urinary tract symptoms (LUTS) in 40% to 70% of men aged ≥60 years. With the aging society and increasing attention on quality of life, there is a growing interest in BPH treatment.^[1]^ Patients with BPH prefer to be treated because the symptoms affect their quality of life, and they can have LUTS, such as feeling of incomplete bladder emptying, frequent urination, intermittency, urgency, weak stream, straining, and nocturia due to BPH.^[2]^ A community-based study in Korea reported that BPH prevalence is 36% in individuals in their 60 seconds, 43% in those in their 70 seconds, and 53% in those in their 80 seconds, and the age-adjusted prevalence was 42%, which showed an increase compared to those in previous studies.^[3]^

The BPH can be divided into mild (0–7 points), moderate (8–19 points), and severe (20–35 points) according to the International Prostate Symptom score (IPSS). When BPH symptoms are moderate or severe, medication administration is recommended as the primary treatment.^[4]^ Medications include alpha blocker, 5-alpha reductase inhibitor, anticholinesterases, a combination of alpha blocker, and 5-alpha reductase inhibitor, and a combination of alpha blocker and anticholinesterases. Surgical treatment is recommended for patients with LUTS who do not improve despite using medications or who complain of severe LUTS that affect the activities of daily living.^[1]^ However, these conventional treatments have limitations due to side effects. In the case of alpha blocker, side effects caused by tamsulosin were reported with 6.6% of rhinitis, 4.4% of dizziness, and 2.8% of abnormal ejaculation.^[5]^ In case of 5-alpha reductase inhibitor, the side effects include erectile dysfunction by finasteride in 4.53%, decreased libido in 2.36%, and abnormal ejaculations in 1.78%.^[6]^ Surgical treatment is difficult to perform on those with severe underlying diseases or difficulties in discontinuing medication prescribed for these diseases (e.g., antiplatelet agents, anticoagulants) and in elderly patients, or postoperative side effects may occur.^[4]^ In this regard, there is still a need for safe and effective alternative treatments that can reduce side effects from surgical treatments and can be applied to patients whose LUTS do not improve despite continued medication use. Recently, electroacupuncture (EA) and moxibustion, one of the complementary and alternative medical treatments, have been suggested as adjuvant therapy to improve LUTS due to BPH. Clinical studies were conducted to evaluate the effectiveness and safety of EA in patients with mild and moderate BPH^[7]^ and moderate or severe BPH.^8^,^9^ EA stimulation on BL35 can stimulate the genital nerve.^[10]^ Additionally, EA stimulation on SP6 and BL33 has been reported to have a positive effect on the sacral nerve root, and EA on SP6 in particular has been reported to stimulate S2 myomere through flexor digitorum longus or posterior tibial nerve.^[11]^ Moxibustion has been reported to be effective in treating voiding dysfunction,^[12]^ and a meta-analysis on the effect of moxibustion on BPH showed that the maximum urinary flow rate (Qmax) of patients with BPH increased after moxibustion, prostate volume decreased, and subjective symptoms and quality of life have improved.^[13]^ However, there has been no report on randomized controlled comparative clinical trials for those who have been diagnosed with BPH and prescribed alpha blocker and had undergone EA and EM combination treatment in addition to continuing the medication that has been prescribed. Therefore, in this study, we have planned a randomized controlled clinical trial evaluating the effectiveness and safety of EA and EM cotreatment by performing the cotreatment on those who have been diagnosed with BPH and used alpha blocker, but the IPSS was between ≥8 points and ≤19 points, with complaint of moderate LUTS.

## 2 Materials and methods

### 2.1 Objective

This clinical study aimed to assess the clinical effectiveness of EA and its cotreatment with EM by comparing the average changes in the IPSS of [EA and its cotreatment with EM + alpha blocker group] and [alpha blocker group] in 6 weeks after the start of treatment, and to determine the safety of EA and its cotreatment with EM by examining the side effects after the treatments.

### 2.2 Study design

**Figure 1:**
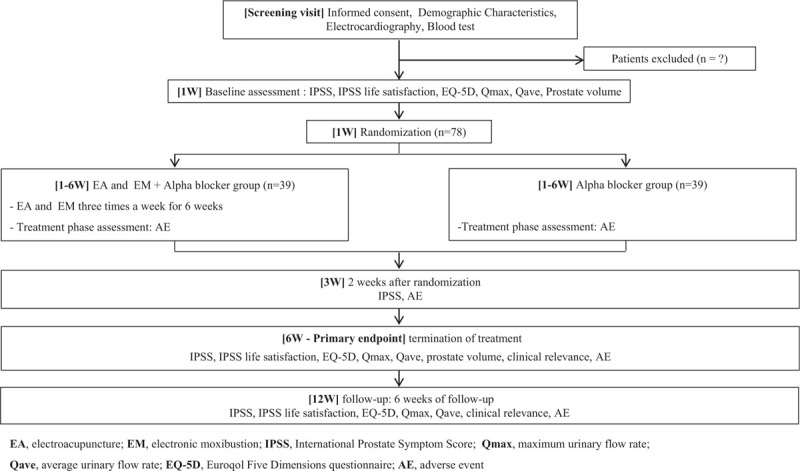
Study flow with outcome assessments.

This clinical trial is a 2-arm parallel-design, randomized, controlled assessor-blinded clinical study. Figure 1 shows a flowchart of this clinical study. A total of 78 subjects who met the inclusion and exclusion criteria (Table 1) of this study were recruited at Dunsan Korean Medicine Hospital of Daejeon University. The volunteers were recruited through advertisements posted on hospitals, bus stops, subway stations, newspapers, and apartment bulletin boards. The recruitment began in November 2019. This clinical study is to be continued for 1 year until November 2020. All subjects are fully informed of the study protocol and provide written informed consent. Additionally, electrocardiogram and blood tests are performed at the screening visits to determine whether the volunteers satisfy the inclusion or exclusion criteria. The subjects who are confirmed to be appropriate for this study are randomized to either groups of [EA and its cotreatment with EM + alpha blocker group] or [alpha blocker] at 1:1 ratio. Intervention shall be conducted within 2 weeks of the screening visit. [EA and its cotreatment with EM + alpha blocker group] continues to use the previously prescribed alpha blocker and visits the institution a total of 18 times for 6 weeks to receive the EA and EM cotreatment. [Alpha blocker group] continues to use the previously prescribed alpha blocker for 6 weeks. On the 12th week, a follow-up examination should be conducted for both groups.

**Table 1:**
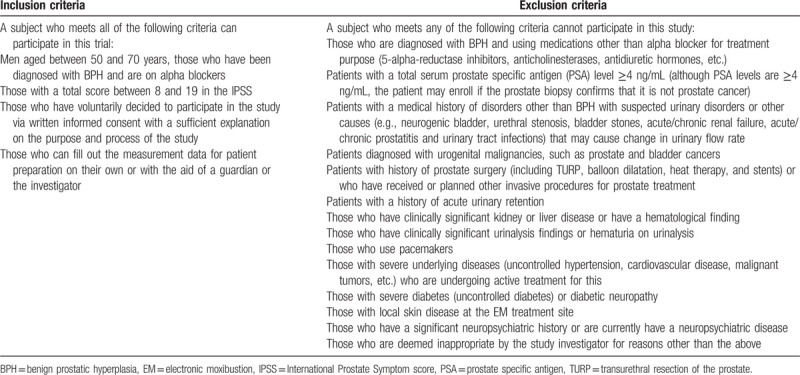
Inclusion and exclusion criteria.

### 2.3 Sample size calculation

Based on previous similar findings and clinical judgment, we assumed that the average change in IPSS before and after intervention (*δ*) was 4.34 and the standard deviation (*σ*) was 5.23 in each group. As a result of applying the significance level of 0.05 and test power of 90%, a minimum of 31 subjects are required in each group, and considering the expected dropout rate of 20%, a total of 78 subjects are required with a minimum of 39 subjects in each group. 

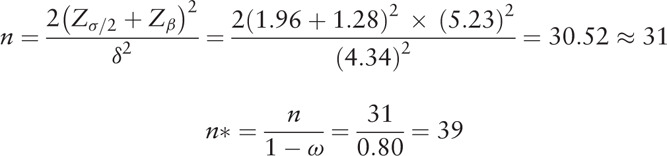



### 2.4 Randomization and blinding

Randomization should not be exposed to subjects and investigators and should be conducted to ensure that treatment assignments are not biased. Statisticians independent of this study randomly assigned 39 subjects to each group using the statistical program SAS version 9.4 (SAS Institute, Inc, Cary, NC), with the same probability of individuals being selected. Statisticians who are not involved in conducting or evaluating this clinical trial will create a randomized table and store the table, ensuring that it is not disclosed.

This study is divided into [EA and its cotreatment with EM + alpha blocker group] and [alpha blocker group], and it is impossible to blind the person who performs the intervention procedure and the subject during the intervention. However, it is designed to be assessor blinded so that the bias can be controlled as much as possible. Investigators who have not performed interventions and randomizations will perform the effectiveness evaluation of the subjects and ensure that the subject does not know what type of treatment the subjects will receive. The investigator simply asks only the evaluation item and details for writing the Case Report Form and performs the evaluation in detail.

### 2.5 Intervention

#### 2.5.1 EA and its cotreatment with EM

Tables 2 ^7^,^8^,^9^,^14^,^15^ and 3^13^,^14^,^16^ summarize the details of the EA and EM cotreatment. Patients in the EA and EM cotreatment group receive a total of 18 EA and EM cotreatments 3 times a week. Disposable sterile stainless steel needles of 0.25 × 40 mm or 0.30 × 60 mm produced by DongBang Medical are used according to the acupuncture point. In 1 procedure, the patient is treated with EA in the prone position and then EA and EM in the supine position. The patient is placed in the prone position, the procedure area is exposed accordingly, and the area is disinfected with 78% alcohol. The investigator locates BL33 to BL35 on both sides and then uses a disposable sterile needle to perform the acupuncture in oblique direction. After inserting the needle, the procedure is performed by applying EA stimulation to BL33-BL35 on both sides for 15 minutes at an intensity of 16 Hz using an EA equipment. After removing the needle, the area is disinfected with 78% alcohol. Then, the patient is placed in supine position, the procedure area is exposed accordingly, and the area is disinfected with 78% alcohol. The investigator locates SP6 to SP9 on both sides and then uses a disposable sterile needle to perform the acupuncture in straight vertical direction. After inserting the needle, the procedure is performed by applying EA stimulation to SP6-SP9 on both sides for 15 minutes at an intensity of 16 Hz using an EA equipment. At the same time as the EA procedure, a sheet of gauze is applied to CV4, and EM cotreatment is performed for 15 minutes with heat stimulus at 45°C. Since different patients have various sensitivities to heat stimulus and tolerance, if a patient complains of heat, a sheet of gauze is applied between the moxibustion and skin. After removing the needle, the area is disinfected with 78% alcohol. All EA and EM cotreatments are performed by a Korean medical doctor with clinical experience of ≥2 years.

**Table 2:**
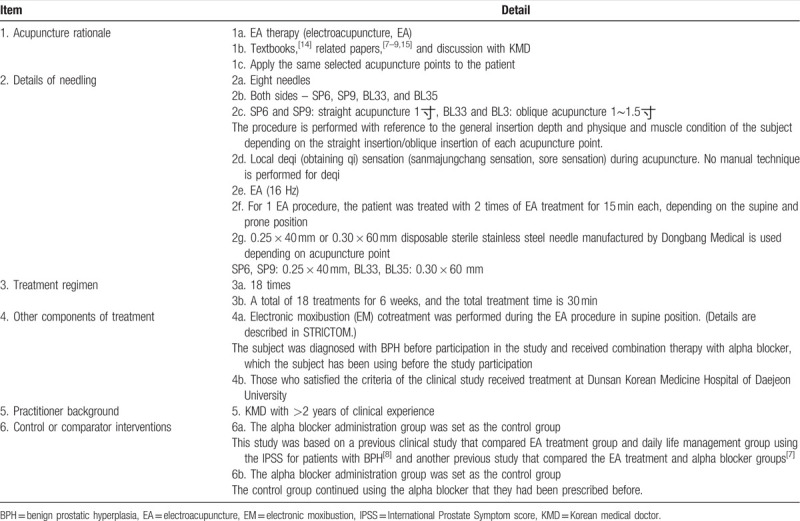
EA treatment details based on Standards for Reporting Interventions in Clinical Trials of Acupuncture (STRICTA) checklist.

**Table 3:**
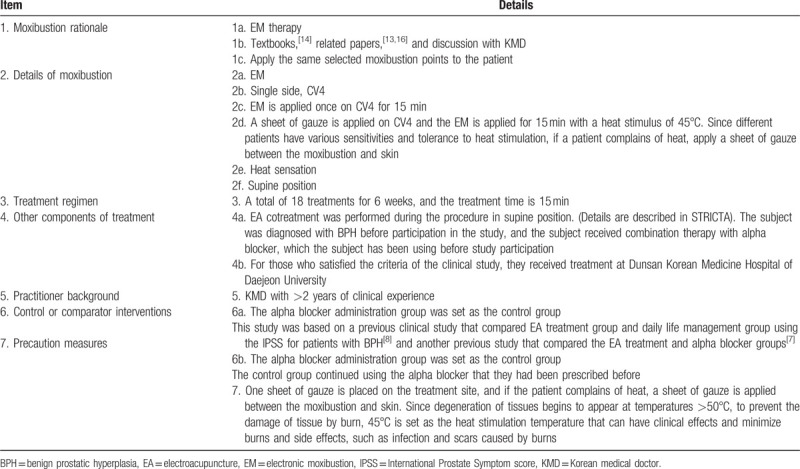
EM treatment details based on Standards for Reporting Interventions in Clinical Trials of Moxibustion (STRICTOM) checklist.

#### 2.5.2 Alpha blocker

Patients in the [alpha blocker group] continue to use the previously prescribed alpha blocker for 6 weeks. During the study period, it is prohibited for the patients to use drugs other than the alpha blocker (such as 5α-reductase inhibitors, anticholinesterases, and antidiuretic hormones) that they were previously using. During the same period, if there is any change or addition to the medication that patients were previously using, the investigator must be notified in advance. After the completion of this clinical study, we will provide up to 3 times of EA and its cotreatment of EM as a compensatory treatment to those who wish to receive the treatment.

#### 2.5.3 Cointerventions

All subjects are prohibited from any other treatments for improving symptoms of BPH other than this study's EA and its cotreatment with EM or alpha blockers that they were previously using. If there is any change or addition to the medication that the patients were previously using, the investigator must be notified in advance. Concomitant medications (including treatment medication in the event of other conditions or adverse events) that are deemed to have no effect on the analysis of the results of this clinical study are permitted under the judgment of the investigators. If it is determined that drugs used by a subject arbitrarily without prior reporting or judgment of the investigators could have a significant impact on the evaluation item of this clinical study, the subject is to be dropped out from the study.

#### 2.5.4 Outcome measures

The evaluation is performed at each of the baseline, the 3rd week (3W), 6th week (6W), and 12th week (12W). Table 4 shows the evaluation schedule. The primary effectiveness endpoint is the average change in the total score of the IPSS on the 6th week. IPSS scores range from 0 to 5 for the following categories: feeling of incomplete bladder emptying, frequent urination, intermittency, urgency, weak stream, straining, urinary hesitancy, and nocturia. According to the severity of symptoms, the scores imply the following symptoms level of the seven items: mild (0–7), moderate (8–19), and severe (20–35).^[1]^ IPSS quality-of-life assessment measures the quality of life of patients with BPH.

**Table 4:**
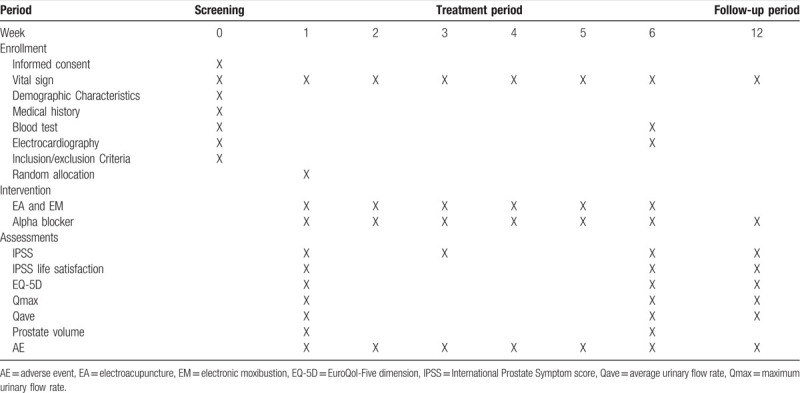
Schedule of treatment and outcome measurements.

The secondary endpoints are the average change in the total score of the IPSS on the 3rd and 12th weeks, clinical correlations, IPSS quality of life assessment, EuroQol-Five dimensions (EQ-5D), maximum urinary flow rate (Qmax), average urinary flow rate (Qave) on the 6th and 12th weeks, and prostate size on the 6th week.

Clinical correlations are assessed using the minimum difference that a patient can feel the clinical effectiveness, namely, the Minimal Clinically Imported Differences (MCID).^[17]^ According to the results of this study examining the clinical correlation of IPSS, it showed that the minimal clinically important difference of IPSS was 3 points for 4 to 6 weeks.^[18]^ In this study, the clinical correlation compares the proportion of subjects whose IPSS has decreased by more than 3 points in each [EA and its cotreatment with EM + alpha blocker group] and [alpha blocker group].

The EQ-5D is the most widely used questionnaire to assess the quality of life. The subjects are required to select the most appropriate sentences under each of the 5 items according to their health status. The selected sentences are coded as numbers 1, 2, and 3 from positive being number 1 and the combination of 5 numbers indicate the health status of the subject.^19^,^20^

The urinary flow rate test is a noninvasive test that provides useful information on urination function by measuring the voided volume per hour. If the urinary flow rate test result is found to be abnormal, bladder outlet obstruction, or detrusor dysfunction can be suspected.^[4]^ Normal Qmax is generally 20 to 25 mL/s, and if the Qmax is <15 mL/s, bladder outlet obstruction can be suspected.^[1]^

The size of the prostate is a factor that affects the clinical progression of BPH and its response to treatment^21^,^22^ and one of the factors that can predict the progression of BPH.^[1]^ Studies show that the tendency to gradual deterioration of IPSS and Qmax were observed over time when the prostate sizes were large and the prostate-specific antigen levels were high,^23^,^24^,^25^ and the progression of BPH can be assessed by measurable factors, such as gradual deterioration of IPSS, decreased Qmax, increased incomplete emptying, and increased prostate size.^[1]^ In this study, physicians measured the prostate size by abdominal ultrasonography. We requested an external radiology department to perform the examination to increase the accuracy of the test.

### 2.6 Adverse events

In this study, adverse events are defined as undesirable and unintended symptoms or diseases that develop after treatments in this clinical study process and are not necessarily causative with this clinical study. Adverse events are assessed based on the vital signs, medical history, and clinical evaluations at each visit. We train the investigators to inform subjects about any possible adverse events after the intervention and report and record all symptoms in the Case Report Form after the intervention. If a serious adverse event (SAE) occurs during the study, the treatment of the subject is temporarily suspended in the clinical trial, and if continuous treatment is deemed to be dangerous to the subject and if the adverse event is related to the intervention, the subject's participation in the clinical trial is permanently discontinued.

### 2.7 Statistical analysis

The data in this clinical trial are analyzed in the forms of a per-protocol group and a full analysis set group. The per-protocol group analyzes the subject group who participated at least 75% (≥14 times of EA and its cotreatment with EM) of the study as specified in the study protocol with endpoint measurements performed and has no violation of the study protocol. The full analysis set group analyzes all subjects except those excluded with valid reasons and minimizes the subjects who are excluded from the analysis among randomized subjects.

All statistical analyses shall be performed using 2-tailed tests in principle, and the significance level is set to 5%. For analysis tool, the statistical program SAS version 9.4 (SAS Institute, Inc) is used. In the case of missing data, the multiple imputation method is used.

Descriptive statistics for each treatment group are presented for variables, such as age, medical history, and medication history, and demographic characteristics, which are basic clinical data of the subjects. To compare each group, in analyzing continuous data, we 1st provide the mean and standard deviation, the independent *t* test or Wilcoxon rank sum test is used for analysis, and a 95% confidence interval is presented if necessary. In analyzing categorical data, we provide the frequency and percentage and then used the Chi-squared test or Fisher exact test.

The primary effectiveness endpoint is the average change in IPSS in 6 weeks against the baseline value, and analysis of covariance is performed with the IPSS value and age as the covariates and each treatment group as the fixed factor. Analysis of the secondary effectiveness endpoint is performed in the same manner as the primary effectiveness endpoint. Paired *t* test or Wilcoxon signed-rank test is performed for the primary and secondary effectiveness endpoints to analyze the difference in the measured values before and after the treatments in each group. To test the difference of trend change per visit, repeated-measures analysis of variance is used, and Dunnett procedure with multiple comparison correction is performed.

The safety assessment is performed primarily by investigators with the analysis on the frequency of the occurrence of AE and SAE suspected of the correlation with the treatment. The frequency of the AEs correlated to the study and those that are not correlated are recorded and presented as descriptive statistics. Adverse events are collected through the observations of investigators and symptoms of patients.

### 2.8 Withdrawal and dropout

The investigator records the completion of the study by all participants in the clinical study and records the reasons for the discontinuation of the procedure, if any. If any violation of the exclusion criteria is found during the study, SAE occurs, or the clinical study is difficult to continue due to the adverse events, the study shall be discontinued.

### 2.9 Ethics and monitoring

This study is based on the Declaration of Helsinki and conducted according to the Korean Clinical Practice Guidelines. This study was approved by the Institutional Review Board of Dunsan Korean Medicine Hospital of Daejeon University (DJDSKH-19-BM-13) and registered on the Clinical Research Information Service (identifier, KCT0004411). All subjects are fully briefed of the study protocol and provided written informed consent. Subjects may withdraw from the trial at any time even after having provided their consent, and all information regarding the subjects and study process are kept confidential. After the completion of this study, an independent investigator analyzes the data for statistical analysis, and a 3rd-party independent investigator conducts monitoring of the process of the clinical study and study data. The findings of this clinical study will be published in the peer-reviewed journal.

## 3 Discussion

This clinical study aimed to assess the clinical effectiveness and safety of EA and its cotreatment with EM in the treatment of patients with moderate BPH. While a number of clinical studies have been published to assess the effectiveness and safety of EA and moxibustion treatment of patients with BPH,^7^,^8^,^9^,^13^ none of them have reported regarding patients with BPH who continue to use prescribed alpha blockers together with EA and its cotreatment with EM. Therefore, this study seeks to assess the clinical effectiveness and safety of the EA and its cotreatment with EM in patients who are diagnosed with BPH and using alpha blockers but still have an IPSS of 8 to 19 points.

For those who have been diagnosed with BPH and prescribed alpha blockers, the inclusion and exclusion criteria of this study are examined to determine whether the subjects can participate in this clinical trial. To prevent SAE that may result from the EA and its cotreatment with EM, the medical history, electrocardiogram, and blood tests are analyzed, and subjects with clinically significant findings are excluded. However, this study does not preclude the possibility of nonspecific impacts, such as placebo or treatment expectations, as differences in the treatment methods in the interventions mean that it is impossible to blind the investigators and subjects. Despite such limitations, this is the 1st clinical trial in which patients diagnosed with BPH are evaluated for the clinical effectiveness and safety of EA and its cotreatment with EM. This study will provide useful information for evaluating the clinical effectiveness and safety of EA and its cotreatment with EM for improving LUTS caused by BPH.

## Author contributions

**Conceptualization:** Hyo Bin Kim, Young Il Kim.

**Data curation:** Ojin Kwon, Young Eun Choi.

**Formal analysis:** Ojin Kwon.

**Funding acquisition:** Chang-Hyun Han.

**Investigation:** Hyo Bin Kim.

**Methodology:** Ju Hyun Jeon, Eunseok Kim, Jin Youp Kim, Changsop Yang.

**Project administration:** Young Eun Choi, Changsop Yang.

**Software:** Ojin Kwon.

**Supervision:** Young Il Kim, Chang-Hyun Han.

**Validation:** Young Eun Choi.

**Writing – original draft:** Hyo Bin Kim.

**Writing – review & editing:** Ju Hyun Jeon.

Chang-Hyun Han orcid: 0000-0003-4285-3063.

## Supplementary Material

Supplemental Digital Content
